# A Metagenomic Approach to Cyanobacterial Genomics

**DOI:** 10.3389/fmicb.2017.00809

**Published:** 2017-05-09

**Authors:** Danillo O. Alvarenga, Marli F. Fiore, Alessandro M. Varani

**Affiliations:** ^1^Faculdade de Ciências Agrárias e Veterinárias, Universidade Estadual Paulista (UNESP)Jaboticabal, Brazil; ^2^Centro de Energia Nuclear na Agricultura, Universidade de São Paulo (USP)Piracicaba, Brazil

**Keywords:** bioinformatics, microbial ecology, genome assembly, metagenome binning, symbiosis, microbial consortia, oxyphotobacteria

## Abstract

Cyanobacteria, or oxyphotobacteria, are primary producers that establish ecological interactions with a wide variety of organisms. Although their associations with eukaryotes have received most attention, interactions with bacterial and archaeal symbionts have also been occurring for billions of years. Due to these associations, obtaining axenic cultures of cyanobacteria is usually difficult, and most isolation efforts result in unicyanobacterial cultures containing a number of associated microbes, hence composing a microbial consortium. With rising numbers of cyanobacterial blooms due to climate change, demand for genomic evaluations of these microorganisms is increasing. However, standard genomic techniques call for the sequencing of axenic cultures, an approach that not only adds months or even years for culture purification, but also appears to be impossible for some cyanobacteria, which is reflected in the relatively low number of publicly available genomic sequences of this phylum. Under the framework of metagenomics, on the other hand, cumbersome techniques for achieving axenic growth can be circumvented and individual genomes can be successfully obtained from microbial consortia. This review focuses on approaches for the genomic and metagenomic assessment of non-axenic cyanobacterial cultures that bypass requirements for axenity. These methods enable researchers to achieve faster and less costly genomic characterizations of cyanobacterial strains and raise additional information about their associated microorganisms. While non-axenic cultures may have been previously frowned upon in cyanobacteriology, latest advancements in metagenomics have provided new possibilities for *in vitro* studies of oxyphotobacteria, renewing the value of microbial consortia as a reliable and functional resource for the rapid assessment of bloom-forming cyanobacteria.

## Introduction

Next generation DNA sequencing technologies became widely available in the middle 2000's, acting synergistically with advances in computer sciences and instigating a revolution in genomics (Koboldt et al., 2013). These technological advancements are much faster in acquiring data and enabled the analysis of much larger biological datasets than possible with the methodology of Sanger et al. (1977), which had become the standard DNA sequencing method for over three decades. The bioinformatics community has kept up with these advancements and developed a considerable number of computer software for analyzing this ever-growing amount of biological information. Following these new methods, not only has genome characterization become quicker, but large-scale projects involving genomics and metagenomics have also become feasible.

**Figure 1:**
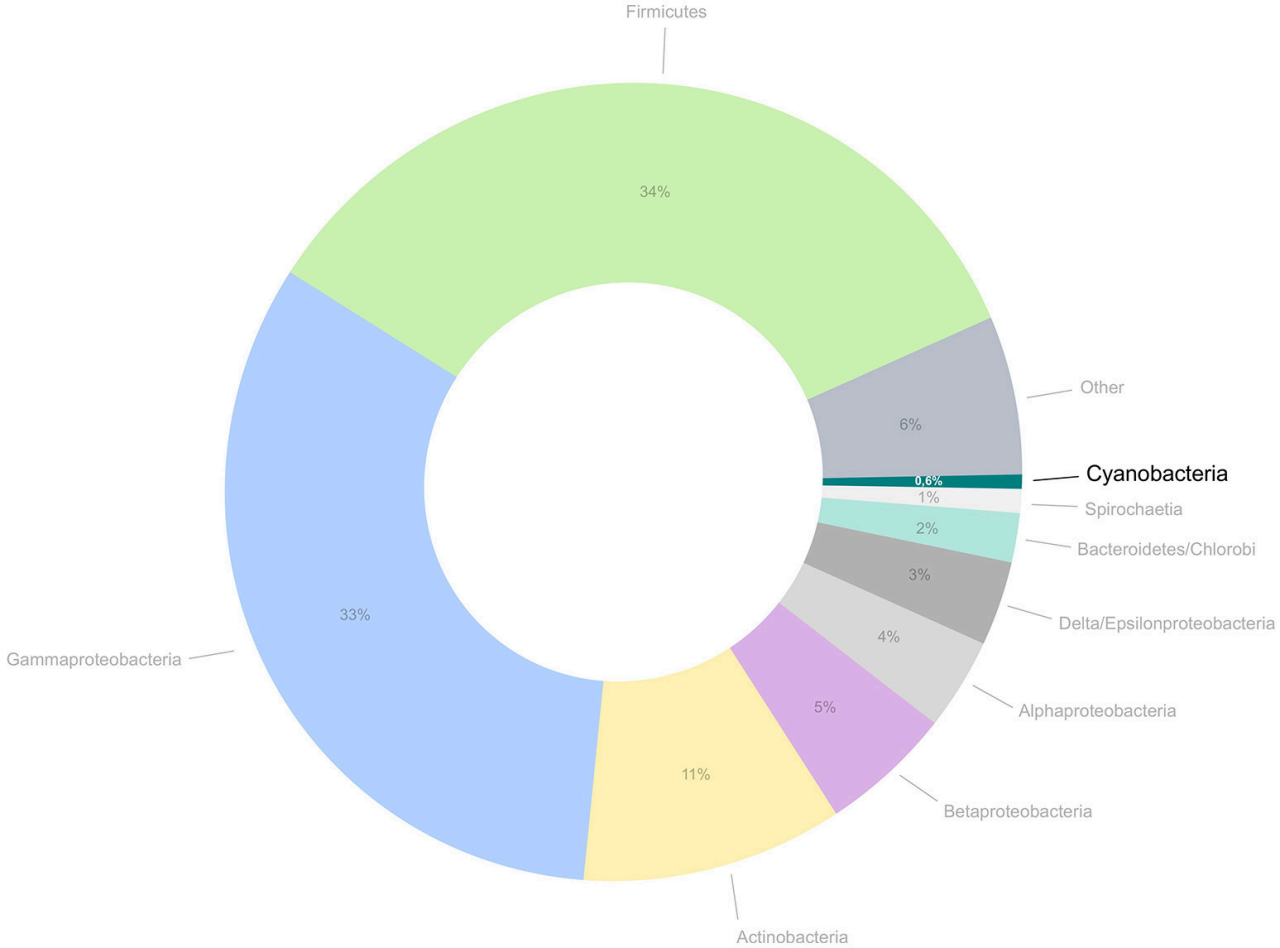
**Proportion of public genomes from cyanobacteria in comparison to the total number of genomes currently available for bacteria and archaea**. The number of cyanobacterial genomes amounts for approximately 0.6% of all prokaryotic genomes available at this moment.

Despite the huge advances recent technologies have brought to microbiology, several obstacles still need to be overcome for a broader characterization of the domain Bacteria, as several phyla are still insufficiently covered by genomics. The field of cyanobacterial genomics holds an interesting example of a research subject that has been moving forward at a pace that is relatively slower than currently observed for some of the other bacterial phyla. Although an increasing number of laboratories around the world are interested in entering this research field, the availability of genomic sequences from cyanobacteria is still relatively low (Figure 1). Therefore, cyanobacteria are severely underrepresented in genomic databases when compared to other bacteria, and even archaea. Additionally, the currently available cyanobacterial genome databases are still lacking in taxonomic, environmental, and geographical diversity, thus providing an incomplete picture of this phylum.

The phylum Cyanobacteria groups oxygenic phototrophic bacteria, or oxyphotobacteria, the likely descendants of the pioneers of oxygenic photosynthesis (Fischer et al., 2016; Shaw, 2016). Most research on cyanobacteria is guided by evolutionary, ecological, ecotoxicological, biochemical, and taxonomic concerns (for a review on common investigation topics regarding cyanobacteria see Sciuto and Moro, 2015). Cyanobacteria were amongst the earliest organisms on the planet and synthesized important molecules for primitive life (Banack et al., 2012; Schirrmeister et al., 2016). Later, microorganisms of this phylum were responsible for oxygenating Earth's atmosphere (Shih et al., 2017) and originating chloroplasts (Alda et al., 2014). Currently, oxyphotobacteria (also known as blue-green bacteria) are important primary producers, with some taxa capable of fixing both atmospheric carbon and nitrogen (Hartmann et al., 2014; Karlson et al., 2015). Some of these organisms cause ecological disturbances after blooming in natural and eutrophic waters, an event that is becoming more frequent as the climate changes (Paerl and Otten, 2013; Costa et al., 2016; Visser et al., 2016). In addition, cyanobacteria are a subject of scientific investigation in regard to their production of toxic and non-toxic secondary metabolites (Merel et al., 2013; Micallef et al., 2015; Pearson et al., 2016). Their troubled taxonomic history has also left an ongoing need for revisiting the systematics of this phylum (Hoffmann et al., 2005; Komárek et al., 2014).

With the help of genomic methods, these topics are now under deeper scrutiny. It is now easier to gather information for understanding the evolution, organization, and distribution of genes involved in cyanotoxin biosynthesis (Stucken et al., 2010; Dittmann et al., 2013; D'Agostino et al., 2016), as well as investigating their possible ecophysiological functions and evolutionary advantages (Holland and Kinnear, 2013; Neilan et al., 2013); to acquire a better view of secondary metabolism and discover new molecules (Baran et al., 2013; Méjean and Ploux, 2013; Calteau et al., 2014; Dittmann et al., 2015; Moss et al., 2016); to verify the structure of DNA packaging (Lehmann et al., 2014); to better comprehend the relationship between morphology and genetics (Dagan et al., 2013; Gonzalez-Esquer et al., 2016); to advance the evolutionary history of the phylum and early life on Earth (Cardona et al., 2015; Harel et al., 2015; Schirrmeister et al., 2015); to study geological and biogeochemical alterations that occurred concurrently with changes in microbiota (Kaufman, 2014; Hamilton et al., 2016); and to refine systematics (Komárek et al., 2014; Thompson et al., 2015). Metagenomics has also made important discoveries, advancing our knowledge on macroevolution (Zaremba-Niedzwiedzka et al., 2017) and revealing a huge diversity yet to be explored (Hug et al., 2016), including the melainabacteria, the closest known relatives of oxyphotobacteria (Di Rienzi et al., 2013; Soo et al., 2014).

These investigations demonstrate that research on cyanobacteria gathers valuable knowledge on a broad range of subjects, and that genomics is well-suited for achieving breakthroughs. Therefore, the small number of available genome sequences for this phylum does not necessarily reflect irrelevance of the topic or lack of interest in either cyanobacteria or genomics; rather, it is likely a consequence of peculiarities inherent to current research methods on cyanobacteria. Difficulty in obtaining pure cyanobacterial cultures, technical challenges in the study of mixed cultures, and the very idiosyncrasies of cyanobacterial genomes are the main factors contributing to the complexity of this research theme, and they must be better understood and adequately addressed for more genomes of these microorganisms to become available. This review explores these factors and highlights joint genomics/metagenomics workflows that aim to overcome some of the challenges commonly found in cyanobacteriology.

## Current state of cyanobacterial genomics

In 1996, *Synechocystis* sp. PCC 6803 became the first cyanobacterium to have its genome published (Kaneko et al., 1996). Yet, more than 20 years later, just a few over 400 cyanobacterial genomes are available in public databases, a number that pales in comparison to more than 30,000 complete genomes available for strains classified in 50 bacterial and 11 archaeal phyla (Land et al., 2015). Additionally, most available cyanobacterial genomes were retrieved from sea or freshwater strains deposited at the Pasteur Culture Collection (PCC) in Paris, France. Although the importance and quality of the PCC is undeniable, this predominance means that public databases are lacking in geographical diversity, as the majority of cyanobacterial strains deposited in the PCC are European. Also worrisome is the fact that some cyanobacterial taxa are overrepresented. As of this moment, 166 genomes were obtained from *Prochlorococcus* spp., among which 45 belong to a single species, *P. marinus*. Thus, the current public cyanobacterial genomes dataset is a biased sample of natural diversity in this phylum. Nevertheless, this dataset has helped to uncover fundamental information about these microorganisms.

The most recent ancestral genome for the cyanobacteria was estimated as having approximately 4.5 Mb and somewhere between 1,678 and 3,291 genes, with only around 4–6% remaining exclusive to the genomes of modern cyanobacteria, which have innovated in sequences for filament development, heterocyte differentiation, diazotrophic metabolism, and symbiotic competence (Larsson et al., 2011). Nonetheless, some of these innovations, like multicellularity, appear to have been acquired and lost many times during the evolutionary history of this phylum and may have been inherited from its common ancestor (Schirrmeister et al., 2011). By looking at the complete genomes published, it can be observed that the genetic material in cyanobacteria is composed of one or two chromosomes (Wang H. et al., 2012), ranging from 1.4 to 8.2 Mb (Meeks et al., 2001; Zehr et al., 2008), with up to 12 plasmids (Hirose et al., 2015), and occasionally an incision element (Thiel et al., 2014). Nevertheless, molecular analyses of the number of chromosomes in cyanobacteria indicate this phylum also presents polyploidy, so that some cyanobacteria contain up to 218 chromosomes during exponential growth (Griese et al., 2011). Akinetes may contain up to 450 chromosomes, most likely due to the necessity of a fast comeback for metabolic activities and cell division after dormancy (Sukenik et al., 2012).

Genomic content can be either the result of neutral processes or the reflection of adaptation to the different conditions an organism is subjected (Barrick et al., 2009; Koonin, 2009; Tenaillon et al., 2016). Basically, two adaptation strategies can be inferred from genomic analyses of cyanobacteria; broad adaptation potential through the increase of gene families as a result of genomic expansion, and elimination of genes that are dispensable for adaptation to a certain niche via mechanisms of genomic reduction (Larsson et al., 2011). Selection pressures may cause changes in genetic factors such as genome size, G-C percentage, gene number, and evolutionary rates. While cyanobacteria may develop individual strategies for interacting with the environment, several of their systems are globally conserved (Simm et al., 2015). Similar to what occurs in other microorganisms, a set of essential genes is found in cyanobacteria presenting considerably high conservation and resistance to horizontal transfer. This conserved gene set, or their core genome, consists mostly of sequences coding for complex protein structures and indispensable biochemical pathways (Shi and Falkowski, 2008; Larsson et al., 2011).

Non-essential genes, part of the accessory genome, are more frequently subject to horizontal gene transfer, which plays an important role in generating molecular diversity in cyanobacteria (Zhaxybayeva et al., 2006). Genome plasticity in these microorganisms is evidenced by the broad distribution and hypervariability of mobile genetic elements, mainly represented by insertion sequences, which can amount up to 10.95% of some genomes (Lin et al., 2010). The relatively high amount of repeated sequences found in the genomes of a number of cyanobacteria is a limiting factor for bioinformatic assembling, even after increasing sequencing depth. Therefore, these repeated sequences directly impact the cyanobacterial genome completeness during assembly. As is well known, large amounts of repeats deliver great challenges for assembling algorithms (Wang H. et al., 2012) and hence are an important factor to explain why approximately 90% of the genomes thus far available are permanent or temporary drafts (Land et al., 2015). Even for cyanobacterial strains in axenic cultures, genome sequencings with high depth and varying library strategies may prove insufficient for reconstructing chromosomes and plasmids into single sequences or even attending the minimum assembly quality for acceptance into the NCBI RefSeq genomes database (N50 above 5,000, L50 under 200, and less than 1,000 contigs) (Tatusova et al., 2013, 2015).

Techniques based on polyphasic taxonomy were introduced to cyanobacterial systematics in an attempt to overcome shortcomings brought by traditional emphasis on morphological features (Vandamme et al., 1996; Komárek, 2005, 2016). Genomics is currently the most promising framework for correcting mistakes caused by traditional taxonomics and clearing out the complicated evolutionary relationships of several polyphyletic taxa persisting in cyanobacterial classification. Potentially, even misleading errors caused by horizontal gene transfer or other processes that obscure the phylogenetic signal could be solved by phylogenomics (Kauff and Büdel, 2011). While the refinement of polyphasic taxonomy is still being discussed (Palinska and Surosz, 2014; Mishra et al., 2015), comparative genomics is its next logical step, and phylogenomics and synapomorphy analyses may redefine our understanding of the evolution of cyanobacteria (Gupta, 2009; Gupta and Mathews, 2010; Komárek et al., 2014).

Phylogenetically, the unbalanced availability of genomic sequences from cyanobacteria causes an unsatisfactory representation of their genomic potential. This prevents the expansion of knowledge of the molecular biology of the phylum, since sequences from neglected taxa may bring to light answers to meaningful questions (Richards, 2015). Sequencing genomes from cyanobacteria of lesser known taxa allows not only increasing knowledge of the molecular genetics of the phylum, but also of the evolution and diversity of aspects such as morphology, photosynthesis, secondary metabolism, and endosymbiosis (Dagan et al., 2013; Shih et al., 2013). There is a clearly observable tendency in more recent genomic projects, to recognize the necessity of studying cyanobacteria from taxa that were poorly investigated or that come from less explored environments. Due to the relatively low number of available genomes from cyanobacteria, applying genomic approaches to some topics was not yet successful on a larger scale. However, even if at this moment there are technical questions preventing a broader use of genomics in cyanobacterial research, genome sequencing is becoming so much faster and cheaper, that it is likely to eventually become a standard procedure.

## Ecological associations involving cyanobacteria

Cyanobacteria present a broad range of metabolic capacities that lead them to perform important ecological roles and to establish mutualistic interactions with a wide variety of organisms. Epi- or endobiotic symbioses between cyanobacteria and eukaryotes such as animals (ascidia, echiuroid worms, midge larvae, sponges), chromalveolata (diatoms, dinoflagellates), fungi (lichens, *Geosiphon*), and plants (cycads, hornworths, liverworts, mosses, *Azolla, Gunnera*) have been documented (Adams, 2000; Bergman et al., 2007; Adams et al., 2013). On the other hand, associations between cyanobacteria and other bacteria or archaea have not received similar attention.

**Figure 2:**
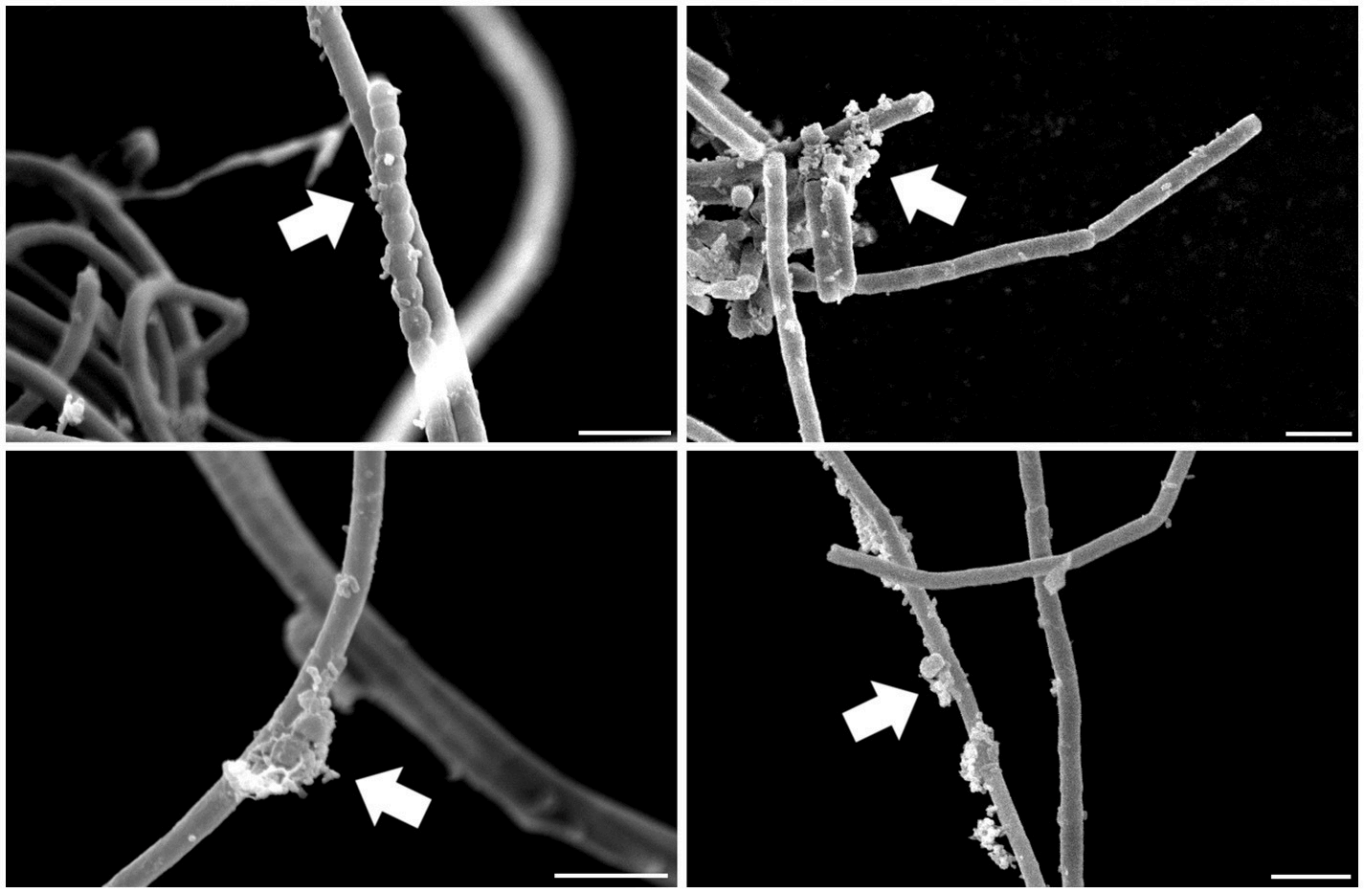
**Scanning electron microscopy of a cyanobacterial strain after standard isolation procedures showing microbes associated with its filaments in a carbon- and nitrogen-free culture medium**. Arrows highlight microbial assemblages attached to cyanobacterial sheaths. Scale: 5 μm.

Several heterotrophic microorganisms benefit from associations with bacteria capable of oxygenic photosynthesis, nitrogen fixation, and biosynthesis of secondary metabolites, characteristics found in many cyanobacteria. Indeed, cyanobacteria often present heterotrophic microbes in symbiotic association with their cells (Figure 2). Some of these associates are in intimate contact with their cell envelope or even reside inside their glycocalyx (Zhubanova et al., 2013). The interacting interface between cyanobacteria and heterotrophic microbes in cyanobacteria-dominated communities could even be considered a “cyanosphere” in analogy to what is observed in plant rhizospheres and phyllospheres.

Associations between cyanobacteria and other microorganisms have been presumed to exist even amongst the oldest known forms of life, engaging in important ecological interactions for billions of years. Stromatolites are a classical example of cyanobacteria-dominated mats supporting highly-developed microbial communities that establish complex interactions (Cohen and Gurevitz, 2006). Exopolysaccharides secreted by cyanobacteria are constantly colonized by microbes and may originate biofilms and microbial mats with high richness and abundance, where autotrophs and heterotrophs find diverse opportunities for interacting (Paerl et al., 2000; Cole et al., 2014). Biofilms dominated by cyanobacteria usually have high nutritional quality and may support large biomasses of primary consumers (Yamamuro, 1999; Nagarkar et al., 2004). Similarly, cyanobacterial blooms occurring in response to eutrophic conditions in water bodies may also be followed by associations with heterotrophic bacteria, several being capable of enhancing cyanobacterial growth (Berg et al., 2009).

In addition to bioavailable carbon and nitrogen, microbes interacting with cyanobacteria may benefit from the secondary metabolites they produce. For instance, some cyanotoxins and cyanopeptides may be degraded and taken up by associated bacteria (Kormas and Lymperopoulou, 2013; Briand et al., 2016). A wide range of chemicals are synthesized by cyanobacteria both under axenic and symbiotic conditions, either in artificial or natural habitats. Molecules with antibacterial, antiprotozoan, antitumor, immunomodulatory, and protease-inhibiting activities have been described, pointing to cyanobacteria as a prolific source for the production of bioactive compounds (Singh et al., 2011). Some authors consider the potential for biosynthesis of secondary metabolites from these organisms as matched only by the potential of myxobacteria and the actinobacterial genus *Streptomyces* (Nunnery et al., 2010). Though the ecological or physiological role of a considerable number of these metabolites is not yet understood, the chemical ecology of this phylum has been an increasingly explored topic (Leão et al., 2012).

## Non-axenic cultures in cyanobacteriology

Especially because of the associations between cyanobacteria and other microorganisms, the process for obtaining axenic cyanobacterial cultures is very challenging. Even with the use of cyanobacteria-specific media, heterotrophs may quickly overcome cells of the target cyanobacterium during isolation, and consequently culture purification becomes a very complex and time-consuming process (Waterbury, 2006). Although several techniques for the purification of cyanobacterial cultures including both mechanical and chemical methods have been published, axenic cyanobacterial cultures are still very hard to achieve, since most methodologies for axenity are somewhat specific to a few strains and have low rates of success (Choi et al., 2008; Sena et al., 2008). Some commonly-employed techniques, such as washing, centrifugation, or filtering are merely capable of reducing the number of unattached microorganisms that are either smaller or larger than the target cells (Vázquez-Martínez et al., 2004; Sena et al., 2008), and they usually cannot remove microbes strongly connected to cyanobacterial sheaths. In certain instances, an axenic culture is considered virtually impossible to obtain due to strong associations between cyanobacteria and symbiont microbes. In addition, fibrous carbohydrate structures in cyanobacterial mucilage may establish firm aggregates with microbes, resulting in strong connections capable of even protecting from antibiotic action (Vázquez-Martínez et al., 2004). Therefore, recalcitrant heterotrophs are a constant concern during the process of isolating cyanobacteria.

Most conventional efforts for the isolation of cyanobacteria result in non-axenic cultures, consisting of microbial consortia composed of a single cyanobacterial species and a number of closely associated non-cyanobacterial organisms, or a unicyanobacterial culture. These cultures are almost like “*in vitro* blooms” in the sense that they provide essential nutrients and conditions for cyanobacteria to massively reproduce and dominate their communities while bringing forward a number of ecological associations with microbes that are potentially host-specific, as commonly observed in environmental blooms (Pope and Patel, 2008; Berg et al., 2009; Bagatini et al., 2014). Most research labs keep non-axenic, unicyanobacterial cultures for routine work, and only dedicate time for trying to achieve an axenic culture when a specific strain is scheduled to be studied in further detail, e.g., for genomic sequencing. Furthermore, traditional methods for evaluating axenity in cyanobacterial cultures may be misleading, as they check for the persistence of cultured bacteria while ignoring uncultured symbionts, causing a mixed culture to be mistaken for an axenic one (Heck et al., 2016).

Another fact adding to the routine use of non-axenic cultures in cyanobacteriology is that these microbes were initially studied in botany, and for legacy reasons the International Code of Nomenclature for algae, fungi, and plants (Botanical Code) (McNeill et al., 2012) is still used for describing cyanobacteria in addition to the International Code of Nomenclature of Prokaryotes (Prokaryotic Code) (Parker et al., 2015). Even today, decades after cyanobacteria were proved to be prokaryotes and not algae, very few cyanobacteria have been described under the Prokaryotic Code (Oren, 2011), and one of the reasons for this is that the Botanical Code does not require axenic cultures as type material, unlike the Prokaryotic Code. As knowledge of microbial ecology advances, the requirement of axenic cultures in the Prokaryotic Code may come to be viewed as unnecessary, unrealistic, or anachronistic in the advancement of microbial systematics, since the great majority of microorganisms are likely to remain uncultured in the near future, due not only to the lack of knowledge of their physiological demands, but also to the incredible amount of work that would be necessary for culturing them, even if it were possible to remove these organisms from their ecological context.

Discussions about the Prokaryotic Code validating cyanobacterial names validly published under the Botanical Code (Pinevich, 2015) and adopting alternative type materials for the description of new microbes (Chun and Rainey, 2014; Sutcliffe, 2015; Whitman, 2015, 2016) are still in the beginning, thus it is likely the current practices will still be kept for quite some time. It is important nevertheless to also include in this discussion the availability of mixed cultures. Most botanical collections that accept cyanobacterial samples require either dried biomass in exsicata or liquid cultures preserved in formaldehyde, which make samples unsuitable for further work. Microbiological collections, on the other hand, are better equipped to receive *in vitro* microbial consortia, but typically only accept axenic cultures. Consequently, the only available option to obtain non-axenic cyanobacterial cultures mentioned in publications is to ask authors directly. The expansion of type material possibilities should therefore be accompanied by official avenues for depositing microbial consortia, which would greatly favor experiment reproducibility.

By using non-axenic cultures, it is possible to significantly speed up research and achieve breakthroughs faster, since the results are obtained drastically quicker when the time that would be necessary for removing the last remaining associated microbes is eliminated. In addition, if the culture is transferred to fresh media on a frequent basis, the relative abundance of cyanobacterial cells is maintained at higher levels than cells of other individual species in the community, as the conditions offered by the culture medium are directed toward the necessities of cyanobacterial physiology, which reduces interference from associates.

## Advantages of cyanobacteria in microbial consortia

Microbial consortia are usually obtained from the enrichment of environmental samples, which are based on the investigation of microbial assemblages after exposure to conditions that stimulate the growth of certain microorganisms from within the community. Depending on the research objectives, such an approach may present some advantages over the analyses of raw, unrefined environmental samples. Datasets coming from the high-throughput sequencing of non-axenic cyanobacterial cultures can be very similar to metagenomic sequences obtained from enriched microbial consortia.

Although a mixed culture may be initially seen as an undesired outcome, microorganisms in consortia may act synergistically and become more efficient than axenic cultures for some processes, or even perform complex ecological functions with multiple steps, which are only possible with the co-culturing of distinct populations (Brenner et al., 2008). Microbial consortia also allow researchers to carry out studies on ecological interaction and co-evolution (Brenner et al., 2008). Mutualism, competition, predation/parasitism, commensalism, amensalism, and neutralism play a central role in the modeling of the stability and dynamics of communities in consortia; therefore, co-culturing enables the discovery of complex interaction networks, including contact-dependent or -independent mediating molecules (Faust and Raes, 2012; Song et al., 2014).

In a microbial consortium, cyanobacteria may enhance the conditions for the community to perform a certain task (Zhubanova et al., 2013). Furthermore, some microbial consortia supported by cyanobacteria potentially enable the growth of otherwise uncultured microbes. Most bacteria are not currently subject to growth under culturing conditions (Rappé and Giovannoni, 2003). Among these bacteria, obligatory symbionts depend on nutrients or cell interactions provided by other microorganisms, which explains the low success rates in attempts of axenic culturing (Wilson and Piel, 2013). Consequently, co-culturing has been suggested as an alternative for carrying out *in vitro* studies in previously uncultured bacteria (Vartoukian et al., 2010; Stewart, 2012). Low emphasis has been given to cyanobacteria as culturing partners, as research has focused mostly on other photosynthetic microorganisms, such as chlorophytes (Otsuka et al., 2008). Nonetheless, as a consequence of their extraordinary metabolic capacities, cyanobacteria have great potential for promoting the *in vitro* growth of currently uncultured bacteria.

Whereas high-throughput sequencing of non-axenic cultures delivers a great challenge to the genomic study of specific strains, it also provides the researcher with access to the genomes of its symbiotic microorganisms, allowing for a broader investigation. In contrast to conventional microbial genomics approaches, which assume datasets originating from axenic samples, metagenomics deals with data from mixed samples, composed of distinct microbial populations (McHardy and Rigoutsos, 2010). Metagenomic approaches have proved useful for unveiling the composition, structure, genetics, and metabolism of natural and artificial microbial communities and are appropriate for the evaluation of microbial consortia (Song et al., 2014). Metagenomics also allow to successfully retrieve draft, near-complete, or even complete microbial genomes from mixed samples (Tyson et al., 2004; Sharon and Banfield, 2013; Sangwan et al., 2016).

Metagenomic assembly is more efficient under conditions of lower richness and higher genomic coherence, when it faces lower interference of less relevant sequences (Teeling and Glöckner, 2012), and it is also favored by samples composed of distantly-related species (De Filippo et al., 2012), as commonly found in non-axenic cyanobacterial cultures. In addition, genomes obtained by metagenomics commonly represent a hybrid population genome, i.e., a chimeric sequence constructed with sequences from different strains of the same species. This problem is not found in a cyanobacterial genome retrieved from the metagenome of a non-axenic culture, as isolation and culturing procedures are responsible for ensuring a single, monoclonal strain.

Sequences obtained by this method must be subjected to either pre- or post-assembly separation into groups representing the distinct genomes present in the dataset. This separation, called binning, can be carried out based either on methods relying on comparisons to databases constructed from genome references or on unsupervised algorithms that consider differences in sequence composition and/or the differential profiling of genomic coverage (Drögue and McHardy, 2012; Mande et al., 2012; Albertsen et al., 2013; Alneberg et al., 2014; Nielsen et al., 2014). Metagenomic binning algorithms now enable the identification and retrieval of cyanobacterial genomes among the metagenomes of microbial consortia, and thus allow to bypass requirements for axenic cultures. This strategy has great potential for accelerating genomic research and has been successfully employed in the characterization of cyanobacterial genomes in mixed cultures in some recent work (Grim and Dick, 2016; Uyl et al., 2016).

## Strategies for the genomics of axenic cultures

**Figure 3:**
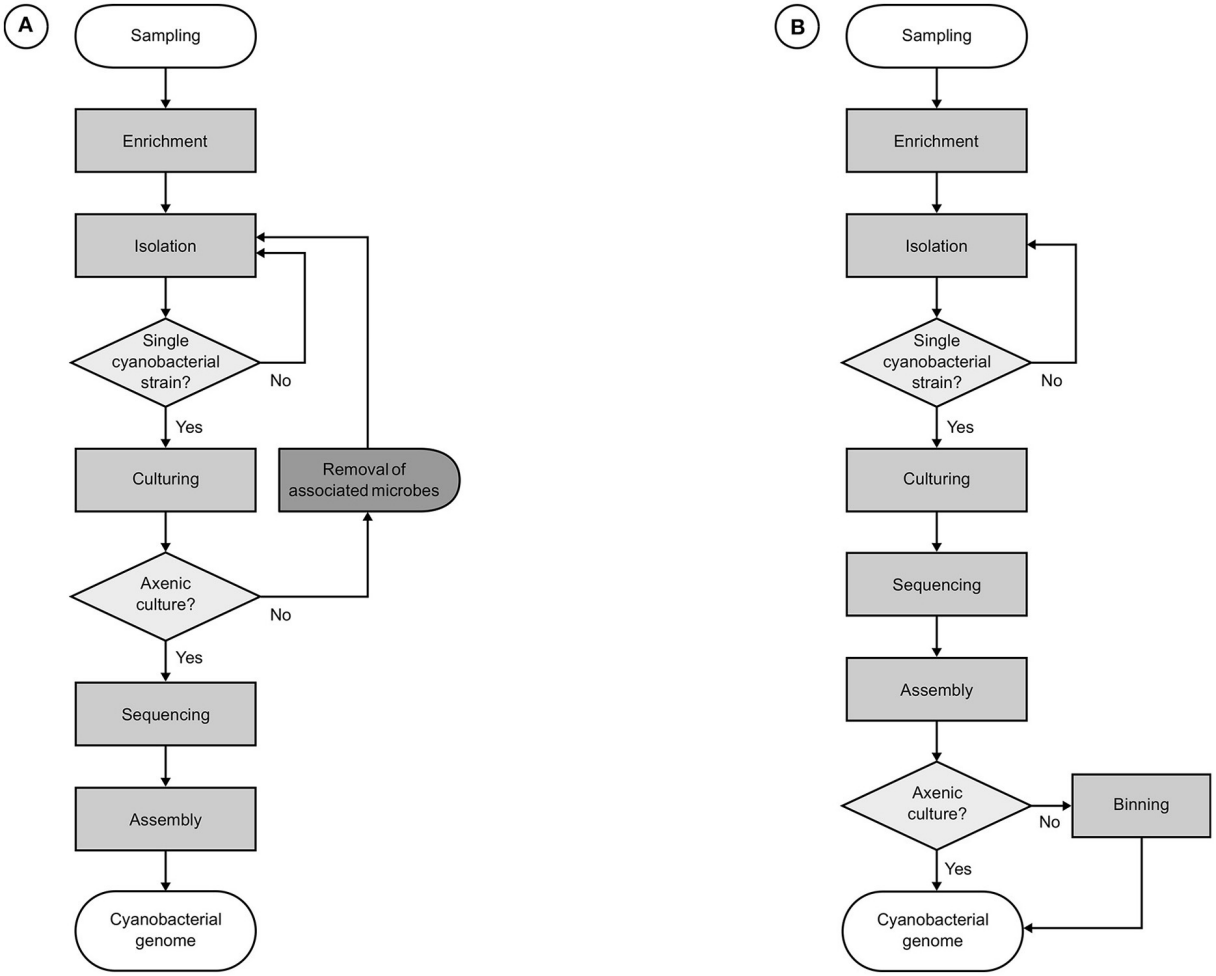
**Workflows for the genomics of cultured cyanobacterial strains. (A)** Usual roadmaps in the traditional approach, relying on the genomic sequencing of axenic cyanobacterial strains. **(B)** Common progression in the genomic characterization of cyanobacterial strains in non-axenic cultures. Both approaches start with the enrichment of an environmental sample by inoculation in cyanobacteria-specific culture media followed by the elimination of the majority of other organisms, leaving only a monoclonal cyanobacterial strain and its most strongly attached associates. For the workflow **(A)**, additional procedures are required for the removal of associated microbes before genome sequencing can be carried out, usually causing major delays. Next, genomic DNA is extracted for the construction of libraries, which are submitted to a high-throughput sequencing platform. Finally, remaining sequencing reads in the filtered datasets are assembled. Workflow **(B)** sidesteps requirements for axenity by performing an additional binning step for the identification and retrieval of cyanobacterial sequences after assembly.

If all associated microorganisms have been removed and a cyanobacterium has been successfully isolated into an axenic culture, a relatively straightforward approach can be employed in its genomic characterization (Figure 3A). Virtually, the only challenges that need to be overcome are those brought by the unique characteristics of the target genome, such as total genomic size, presence and number of extrachromosomal elements, occurrence of repeated regions, and type and abundance of mobile genetic elements, among others. Thus, standard practices in microbial genomics can be readily applied without major modifications, except for some techniques for genome assembly finishing and gap closing, since reference genomes are likely unavailable or too phylogenetically distant. Even when reference genomes have been published, they are likely to present significant rearrangements (Humbert et al., 2013). Consequently, reference assemblies may produce sequences that are neither more accurate nor more complete than those obtained from *de novo* assemblies (Fadeev et al., 2016).

A satisfactory strategy for the genomics of axenic cyanobacterial cultures will be composed basically of read quality control, *de novo* assembly, scaffolding, gap closing, assembly statistics, and genome annotation. First, it is necessary to verify sequencing quality and remove from the reads regions containing bases of low quality scores or unknown bases (represented as “N” in the datasets). Acceptable sequence quality scores depend mainly on sequencing depth and technology, but Phred 20 as the very minimum quality is advised, whereas Phred 28 or higher is recommended for most analyses. We suggest using FastQC (http://www.bioinformatics.babraham.ac.uk/projects/fastqc/) for evaluating sequence quality and PRINSEQ (Schmieder and Edwards, 2011a) for filtering reads, as it is a powerful and flexible software enabling a large number of filtering parameters. FLASH (Magoč and Salzberg, 2011) can be used for merging read pairs with overlapping ends if paired-ends libraries constructed with shorter fragments are available. For genome assembly, SPAdes (Bankevich et al., 2014) currently appears to achieve the best results for most microbial genomes and metagenomes datasets obtained by Illumina sequencing. Platanus (Kajitani et al., 2014) can be employed after SPAdes assemblies for enhancing assembly by carrying out additional scaffolding and gap closing steps. QUAST (Gurevich et al., 2013) is interesting for verifying assembly statistics and comparing results from different workflows. Finally, the assembled genome can be annotated with Prokka (Seemann, 2014), which generates all files necessary for submitting the genome to NCBI, or RAST, which also provides subsystems information (Aziz et al., 2008; Overbeek et al., 2014). We also recommend using the NCBI Prokaryotic Genome Annotation Pipeline (PGAP) (Tatusova et al., 2016), since it is capable of properly identifying and annotating pseudogenes, which are commonly missed by Prokka and RAST.

Although this is viewed as the best scenario for characterizing the genome of a cyanobacterium, this strategy may still come short of producing finished chromosome and plasmid sequences. Nevertheless, draft genomes are suited to a number of analyses, including comparative genomics, as they can contain most of the genetic information of the genome (Humbert et al., 2013; Fadeev et al., 2016). Paired-ends libraries resulting in draft genomes are good enough for several applications, but differing sequencing strategies should be adopted if a finished genome is particularly indispensable for research, including mate-pair libraries, longer sequencing reads, or even BAC cloning.

Single-cell genomics has been recently reviewed as an alternative approach for sequencing cyanobacteria in natural environments (Davison et al., 2015). This strategy consists basically of the capture of a single-cell followed by routines that are similar to the ones employed for axenic strains. Unfortunately, this technology still faces methodological challenges and very few laboratories have access to or familiarity with its methods. In addition, it is also worth considering if it is really desirable to look at the genome of an individual strain and ignore the genomes of its most intimate associates, since ecological interactions are usually an important factor for determining evolutionary influence on genomic content.

## Strategies for the genomics of non-axenic cultures

The main problem with the genomic sequencing of non-axenic cultures is the high number of “contaminating” sequences, which reduce the coverage of the target genome and disturb assembly by introducing increased complexity and noise. In most cases, the conventional genomic approach will be very hard to apply in conjunction with standard practices in the culturing of cyanobacteria, which mostly rely on microbial consortia. For cyanobacteria-dominated microbial consortia, it is more adequate to employ a metagenomics-like approach (Figure 3B). This strategy has to consider additional issues besides the particularities of the target genomes, urging for precautions to be taken both before and after sequencing.

Since microbial consortia contain a mixture of genomes, metagenome sequencing must have higher throughput than genome sequencing in order to obtain satisfactory depth for individual genomes. Several platforms currently provide enough depth and coverage for metagenome sequencing. However, when the cyanobacterial genome is the main target and sequencing is performed either under budget constraints or on a platform that provides longer sequences at the cost of lower depth, it is advisable to ascertain by microbiological methods that the abundance of the cyanobacterium is high enough for its genome to be present among the sequences. In this case, a great deal of caution must be taken in the maintenance of non-axenic cultures so that the abundance of associated bacteria is kept at controllable levels. This is achieved by frequently transferring the culture to fresh media (at a frequency that varies according to the growth rates of the strain) and using mechanical techniques that eliminate the organisms not firmly attached to the cyanobacteria (like streaking and washing, among others). Time spent on further cleaning up a cyanobacterial culture eventually pays off even if it does not result in axenity when faced with limited sequencing.

The diversity of associated microbes can be initially explored in unassembled datasets, so that the relative abundance of operational taxonomic units in the datasets is estimated. FOCUS (Silva et al., 2014) and SUPER-FOCUS (Silva et al., 2015) are useful for quickly verifying diversity in the dataset before assembly and allow the previewing of the relative abundances of the different genomes in the metagenome. Due to the less than satisfactory current state of public databases, most metagenomics software will state there are several different cyanobacterial taxa in the sample instead of a single one, either in the sequencing of a new cyanobacterium or in the resequencing of a known taxon. In the case of unicyanobacterial cultures, the level of phylum should be considered for these analyses, as the still low number of related genomes, if available, most likely do not encompass the whole genomic diversity of the referenced taxon and similar sequences may be found in the genomes of some phylogenetically distant taxa, resulting in the overestimation of cyanobacterial diversity in the sample.

After sequencing, it is fundamental to pre-process the data set for eliminating not only low quality sequences with PRINSEQ as previously mentioned, but also contamination. DeconSeq (Schmieder and Edwards, 2011b) is a very useful tool for removing usual contaminants from metagenomic sequences. If the number of associated microbes is low and reference genomes are available, their sequences can be dealt with in the same way contaminating sequences are, such as mapping sequencing reads to references with Bowtie (Langmead et al., 2009) or BWA (Li and Durbin, 2009) and collecting unmapped reads. If successful, downstream analyses may follow some of the methods employed for axenic cultures. However, if the associated community is richer, binning will need to be carried out either before or after assembly. QUAST can be used for comparing results and evaluating at which point binning has to be carried out for the best results, and CheckM (Parks et al., 2014) is useful for verifying binning reliability.

A relatively intuitive approach for pre-assembly selection of cyanobacterial sequences is to fetch available reference cyanobacterial genomes and format them as a Bowtie database. Reads that are mapped to this database can then be retrieved and *de novo* assembled as if they were originated from an axenic culture. Although this method is commonly brought up by some researchers new to cyanobacterial genomics, most often it only produces meaningful results for taxa that have a considerable number of references available and low genomic variation. However, even in this case, binning the assembled metagenome after assembly will most often achieve better results, including when using a reference-based method. This occurs because of the likely presence of regions exclusive to that novel strain, whose reads may escape selection because of their absence in the reference genomes or of the distance of their evolutionary relationship; reference-based binning of reads before assembly would lead to the removal of a significant amount of unknown sequences, which might otherwise be connected to known sequences into contigs or scaffolds.

Metagenome reconstruction from non-axenic cyanobacterial cultures may also be carried out successfully by using SPAdes for initial assembly and Platanus for additional scaffolding and gap closing steps. After a satisfactory assembly is achieved, metagenome binning should be carried out for separating cyanobacterial sequences and sequences from associated microbes. Binnning is a crucial step in strategies that work with non-axenic cultures, as correct identification of genome sequences ensures the success of downstream annotation. The retrieval of population genomes among metagenomes and their validation has been recently reviewed (Sangwan et al., 2016), and similar methods may apply to the metagenomics of microbial consortia. A considerable diversity of bioinformatic tools is now available for performing metagenomic binning (Drögue and McHardy, 2012; Mande et al., 2012; Sangwan et al., 2016; Sedlar et al., 2017), and yet it is still hard to point to an optimal method for this step (Sangwan et al., 2016), so this step asks for increased attention.

The simplest scenario is presented when sequencing a cyanobacterium with publicly available references. However, if a sequence from a closely related species or genus is not available, references belonging to related taxonomic levels up to phylum might be used for supervised binning (Thomas et al., 2012), which allows for sequences from unreferenced cyanobacteria in unicyanobacterial cultures to be processed by a reference-based strategy. For post-assembly taxonomic assignment, Kraken (Wood and Salzberg, 2014) is usually a good choice, since it has shown good accuracy and very low levels of false positives when compared to other binning software (Lindgren et al., 2016). To facilitate genome retrieval after taxonomic assignment of the assembled sequences by Kraken, we have developed a custom Python script that enables the collection of genome sequences from select taxa among the assembled metagenome based on the Kraken output. This script is freely available at https://www.github.com/danillo-alvarenga/zeuss and can be used for retrieving either the target cyanobacterium genome, the associated community metagenome, or the genome from an associated microbe in particular that has been identified by Kraken.

Depending on the available sequencing data and genomic information, software employing reference-based methods might be more suited to resequencing, while novel taxa could be more adequately addressed by methods based on reference-free algorithms. Reference-based binning is frequently limited by poor databases, which greatly favors unsupervised strategies. Since even genomes from the same species present considerable variation (Humbert et al., 2013), reference-based assembly may not be an optimal methodology even for genomes with references available. Furthermore, modern reference-free binning software can distinguish between genomes until the taxonomic rank of species (Strous et al., 2012; Kang et al., 2015), and thus might be useful even for multicyanobacterial cultures. There are lots of binning software alternatives implementing differing algorithms and methodologies that may be employed in metagenome binning (see Tables 1, 2), and it is hard to favor one over another, but the results of different binning software may be combined and refined for more accurate results (Song and Thomas, 2017). Additionally, some of these methodologies, including those based on differential coverage, benefit from sequencing the cultures at different phases of their growth cycle, such as during log, lag, and early or late stationary phases.

**Table 1:** **Software presently available for unsupervised binning of metagenomes**.

**Program**	**Website**	License^*^	**References**
ABAWACA	https://github.com/CK7/abawaca	BSD	Sangwan et al., 2016
AbundanceBin	http://omics.informatics.indiana.edu/AbundanceBin	proprietary	Wu and Ye, 2011
BinSanity	https://github.com/edgraham/BinSanity	GPL 3	Graham et al., 2017
Canopy	https://bitbucket.org/HeyHo/mgs-canopy-algorithm	GPL 3	Nielsen et al., 2014
CARMA3	http://wwww.cebitec.uni-bielefeld.de/webcarma.cebitec.uni-bielefeld.de	GPL 2	Gerlach and Stoye, 2011
Centrifuge	https://github.com/infphilo/centrifuge	GPL 3	Kim et al., 2016
ClaMS	http://clams.jgi-psf.org	BSD	Pati et al., 2011
COCACOLA	https://github.com/younglululu/COCACOLA	GPL 3	Lu et al., 2017
CompostBin	https://figshare.com/articles/Compost_Bin_Software_and_Data_Sets/717223	proprietary	Chatterji et al., 2008
CONCOCT	https://github.com/BinPro/CONCOCT	BSD	Alneberg et al., 2014
GroopM	https://github.com/ecogenomics/GroopM	GPL 3	Imelfort et al., 2014
LikelyBin	http://ecotheory.biology.gatech.edu/likelybin	proprietary	Kislyuk et al., 2009
MaxBin	https://sourceforge.net/projects/maxbin	BSD	Wu et al., 2014
MBBC	http://eecs.ucf.edu/~xiaoman/MBBC/MBBC.html	proprietary	Wang et al., 2015
MetaBAT	https://bitbucket.org/berkeleylab/metabat	BSD	Kang et al., 2015
MetaCluster	http://i.cs.hku.hk/~alse/MetaCluster	GPL 2	Wang Y. et al., 2012
Metawatt	https://sourceforge.net/projects/metawatt	AFL	Strous et al., 2012
MyCC	https://sourceforge.net/projects/sb2nhri/files/MyCC	proprietary	Lin and Liao, 2016
NBC	http://nbc.ece.drexel.edu	proprietary	Rosen et al., 2010
RAIphy	http://bioinfo.unl.edu/raiphy.php	proprietary	Nalbantoglu et al., 2011
RITA	http://kiwi.cs.dal.ca/Software/RITA	CC 3.0	MacDonald et al., 2012
SCIMM	http://www.cbcb.umd.edu/software/scimm	AL 2.0	Kelley and Salzberg, 2010
VizBin	https://claczny.github.io/VizBin	BSD	Laczny et al., 2015

* *GPL, GNU Public License; BSD, Berkeley Software Distribution License-based; AFL, Academic Free License; AL, Artistic License; CC, Creative Commons. Software without clear, open licensing information was assumed to be proprietary, even when otherwise claimed*.

**Table 2:** **Currently available software for taxonomic assignment of metagenomic sequences**.

**Program**	**Website**	License^*^	**References**
CLARK	http://clark.cs.ucr.edu	GPL 3	Ounit and Lonardi, 2016
Genometa	http://genomics1.mh-hannover.de/genometa	CPL	Davenport et al., 2012
Gottcha	https://github.com/LANL-Bioinformatics/GOTTCHA	GPL 3	Freitas et al., 2015
k-SLAM	https://github.com/aindj/k-SLAM	GPL 3	Ainsworth et al., 2016
Kraken	http://ccb.jhu.edu/software/kraken	GPL 3	Wood and Salzberg, 2014
LMAT	http://sourceforge.net/projects/lmat	proprietary	Ames et al., 2013
MEGAN CE	https://github.com/danielhuson/megan-ce	GPL 3	Huson et al., 2016
Metaphlan	http://huttenhower.sph.harvard.edu/metaphlan	MIT	Segata et al., 2012
MetaPhyler	http://metaphyler.cbcb.umd.edu	proprietary	Liu et al., 2011
MG-RAST	http://metagenomics.anl.gov	BSD	Meyer et al., 2008
MLTreeMap	http://mltreemap.org	proprietary	Stark et al., 2010
mOTU	http://www.bork.embl.de/software/mOTU	GPL 3	Sunagawa et al., 2013
PHY SCIMM	http://www.cbcb.umd.edu/software/scimm	AL 2.0	Kelley and Salzberg, 2010
PhyloPythiaS+	https://github.com/algbioi/ppsp	proprietary	Gregor et al., 2016
PhymmBL	http://www.cbcb.umd.edu/software/phymmbl	proprietary	Brady and Salzberg, 2011
SOrt-ITEMS	http://metagenomics.atc.tcs.com/binning/SOrt-ITEMS	proprietary	Mohammed et al., 2009
Taxator-tk	https://github.com/fungs/taxator-tk	GPL 3	Dröge et al., 2014
TreePhyler	http://www.gobics.de/fabian/treephyler.php	proprietary	Schreiber et al., 2010
TWARIT	http://metagenomics.atc.tcs.com/Twarit	proprietary	Reddy et al., 2012

* *GPL, GNU Public License; BSD, Berkeley Software Distribution License-based; MIT, The MIT License; CPL, Common Public License; AL, Artistic License. Software without clear, open licensing information was assumed to be proprietary, even when otherwise claimed*.

After separation of the cyanobacterium genome, diversity and function analyses can be easily performed on the associated community sequences, as well as functional analyses comparing cyanobacterium and community sequences. The associated community genome can then be automatically annotated with the MG-RAST server (Meyer et al., 2008) or another metagenome annotation software. Finally, manual curation of the cyanobacterial genome after annotation by Prokka, RAST, PGAP or other resource assures the assembly of a single genome. Several pipeline options are available for the analysis and annotation of mobile genetic elements, which have been reviewed elsewhere (Alvarenga et al., in press).

**Figure 4:**
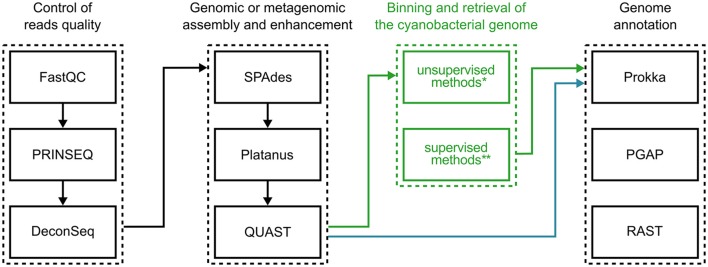
**Suggested software for research on cyanobacterial genomics**. Typical bioinformatics procedures for characterizing cyanobacterial genomes may be divided into three main steps: (1) assessment and filtering of sequencing read quality; (2) assembly of filtered reads; (3) annotation of genomic sequences. When sequencing axenic cultures, genome sequences can be evaluated right after assembly (blue arrows), while the sequencing of mixed cultures depend on binning the assembled consortium metagenome (green arrows). ^*^For a list of software options implementing unsupervised methods, see Table 1. ^**^See Table 2 for a list of software based on supervised methods.

Figure 4 illustrates a possible workflow for the initial genomic investigation of cultured cyanobacteria. The indications provided are based on free and open source bioinformatics tools that are readily available for use on modern GNU/Linux distributions. Please keep in mind that there is now a large diversity of bioinformatics software that can be employed in this effort, and thus many alternative programs could replace the examples given, and some steps could be repeated and reiterated for improved results. Therefore, this suggestion should be taken merely as a starting point.

## Concluding remarks

The assembly of high-throughput reads becomes easier for both genomics and metagenomics when sequencing technologies become capable of generating longer reads. New methods of library preparation, such as Illumina synthetic long reads (McCoy et al., 2014) and developing platforms from the 10x Genomics and Dovetail startups (Eisenstein, 2015), are becoming available for achieving longer reads. Likewise, new generations of sequencing technologies, including Pacific BioSciences SMRT (Rhoads and Au, 2015) and Oxford Nanopore (Laver et al., 2015; Lu et al., 2016), are focused on generating reads of increasing lengths. As sequencing technologies, assembly algorithms, metagenomics tools, and genomic databases advance, so confidence and reliability in mixed-culture assemblies increase, virtually rendering axenity dispensable. Whether this is a desirable outcome or an unfortunate side effect is arguable. If on the one hand it is likely that a smaller number of research labs will keep satisfying traditional microbiology demands and pursue axenity in cyanobacterial cultures, this change may also bring more cyanobacterial genomes to light and advance our comprehension of the molecular biology of this phylum. Nonetheless, it appears to be inevitable that metagenomics becomes a subject of central interest in cyanobacteriology, not only for the study of ecological interactions, but also for advancing knowledge on the genomics and evolution of oxyphotobacteria at an increased pace.

## Author contributions

DA, MF, and AV conceived the review. DA wrote the draft version of the manuscript. MF and AV revised the manuscript critically and provided substantial contributions. All authors read and approved the final version of the article.

## Funding

This work was supported by a grant from the São Paulo Research Foundation (FAPESP) to MF (#2013/50425-8). DA was supported by a FAPESP graduate fellowship (#2011/08092-6) and a post-doctoral fellowship (#2015/14600-5). MF and AV thank the Conselho Nacional de Desenvolvimento Científico e Tecnológico (CNPq) for research fellowships (#310244/2015-3 and #302599/2016-9, respectively).

### Conflict of interest statement

The authors declare that the research was conducted in the absence of any commercial or financial relationships that could be construed as a potential conflict of interest.
